# Oxygen Uptake in Repeated Cycling Sprints Against Different Loads Is Comparable Between Men and Preadolescent Boys

**DOI:** 10.3389/fphys.2022.814056

**Published:** 2022-03-11

**Authors:** Apostolos Theos, Gregory C. Bogdanis, Daniel Jansson, Alan M. Nevill, Aggeliki Papaspyrou, Maria Maridaki

**Affiliations:** ^1^Section of Sports Medicine, Department of Community Medicine and Rehabilitation, Umeå University, Umeå, Sweden; ^2^Umeå School of Sport Sciences, Umeå University, Umeå, Sweden; ^3^School of Physical Education and Sport Science, National and Kapodistrian University of Athens, Athens, Greece; ^4^Faculty of Education Health and Wellbeing, Wolverhampton University, Wolverhampton, United Kingdom

**Keywords:** growth, aerobic, high-intensity exercise, repeated-sprint ability, fatigue

## Abstract

Children recover faster than adults in repeated sprints, but it is unclear if their aerobic responses differ.

**Purpose:**

This study tested the hypothesis that aerobic response (VO_2_) during repeated sprints is greater in preadolescent boys than in men. Further, this study compared normalization with conventional ratio-scaling and scaling with the use of body mass (BM) as a covariate.

**Methods:**

Nine boys (age: 11.8 ± 0.6 years, swimmers) and 11 men (age: 21.7 ± 0.6 years, recreational athletes) performed 10 maximal 6-s cycling sprints separated by 24-s of passive recovery, against two loads (optimum and 50% of optimum). Oxygen uptake (VO_2_) was measured continuously.

**Results:**

Men’s mean power output (MPO) was greater than boys in each sprint, both in absolute (unscaled) values ( *p* < 0.05) and when adjusted for lean leg volume ( *p* < 0.05). Children had lower absolute VO_2_ ( *p* < 0.05) than men, but when it was adjusted for BM or power-output, VO_2_ was comparable between men and boys. Thus, most of the difference in VO_2_ between men and boys was due to body size and power-output differences.

**Conclusion:**

Our results suggest that men and boys have similar VO_2_ during repeated sprints when appropriately adjusted to body mass or power output. Results highlight the importance of using appropriate scaling methods to compare adults’ and children’s aerobic responses to high-intensity exercise.

## Introduction

Children’s everyday activity includes engaging in short bursts of intense physical activity separated by short recovery periods (Bailey et al., 1995). During exercise, children’s physiological responses differ from adults (Van Praagh and Doré, 2002; Ratel et al., 2006). For example, adults generate a greater maximum power output during high-intensity exercise than children (Ratel et al., 2002), while children experience less fatigue (Ratel et al., 2004) and recover faster (Ratel et al., 2002). Therefore, it is evident that growth and maturation affect physiological variables such as oxidative metabolism, muscle fatigability, and aerobic-to-anaerobic power ratio (Ratel and Blazevich, 2017). However, data demonstrating the impact of maturation on the above-mentioned physiological differences are debatable (Bell et al., 1980; Lexell et al., 1992; Dotan et al., 2012; Birat et al., 2018).

Prepubertal children have been compared to well-trained endurance athletes because of their greater net contribution of energy derived from aerobic metabolism in exercising muscle and their ability to resist fatigue (Birat et al., 2018). Aerobic metabolism is one of the performance determinants during repeated sprints (Dupont et al., 2005). Oxygen uptake (VO_2_), both on- and off-kinetics, seem to be related to the ability to maintain sprint performance during repeated sprints (Dupont et al., 2010). Some studies have suggested a child-adult difference in measures of aerobic response during high-intensity exercises (Armon et al., 1991; Leclair et al., 2013; Ratel and Blazevich, 2017). For example, children have shown faster VO_2_ kinetics compared to adults (Leclair et al., 2013). Additionally, the average oxygen cost (i.e., VO_2_ per unit of power) during a single bout of high-intensity exercise is higher in children than adults (Armon et al., 1991), suggesting an increased reliance on the aerobic system. Children’s faster VO_2_ kinetics is possibly the result of faster O_2_ extraction (Leclair et al., 2013), faster phosphocreatine recovery kinetics (Ratel et al., 2008), and differences in type I fiber type distribution (Pringle et al., 2003). Indeed higher muscle aerobic activity favors faster creatine phosphate resynthesis, thus improving recovery following high-intensity exercise (Bogdanis et al., 1995). On a muscle-fiber level, children have a higher proportion of type-I muscle fibers (Hamada et al., 2003). Type-I muscle fibers produce a lower power output but are more fatigue-resistant than type-II muscle fibers (Hamada et al., 2003). However, data on muscle-fiber type composition in healthy children are scarce because of ethical reasons. Therefore, it is unclear whether the proportion of type-I muscle fibers is higher in children (Lexell et al., 1992) or similar to adults (Bell et al., 1980).

Preadolescent children have a reduced capacity to recruit type-II motor units, probably due to their lower motor neuron impulse frequency (Dotan et al., 2012). Motor unit recruitment and the ability to maintain power output during repeated cycling sprints are also related to the selected resistive load (Bogdanis et al., 2007; Matsuura et al., 2011). Our research group has previously shown (Bogdanis et al., 2007) that children’s power output during repeated cycling sprints can be better maintained when they cycle against a resistive load corresponding to the individual optimum force (Fopt). In line with our results, other studies on adult population show similar effects of resistive loads’ influence on power output (Matsuura et al., 2011). In accordance with their reduced capacity to recruit type-II muscle fibers and generate power, children also present lower blood lactate levels during short, high-intensity exercises (Ratel et al., 2004; Falk and Dotan, 2006). Some have reported a greater lactate clearance in children than adults (Beneke et al., 2005), although others report similar lactate clearance compared to adults (Dotan et al., 2003; Dotan and Falk, 2015).

Studies examining differences in VO_2_ between children and adults during high-intensity exercise are limited to single bouts exercise, with no studies examining repeated sprints. Further, the majority of the studies comparing VO_2_ and power output between children and adults use traditional ratio-scaling (ml kg^−1^ min^−1^) to account for body mass (BM) differences, a method that unfairly favors lightweight individuals and should be avoided in child-adult comparisons (Nevill et al., 1992). Instead, scaling models based on linear regression have provided a better statistical fit than ratio-scaling when comparing children and adults (Nevill et al., 1992; Welsman et al., 1996). The method has successfully been used to scale VO_2_ to body size (Welsman et al., 1996) and normalize anaerobic variables such as power output (Carvalho et al., 2011; Armstrong et al., 2019) and isokinetic strength (Carvalho et al., 2012). Although earlier studies compared child-adult differences in oxygen uptake, it is still unclear whether a difference exists.

We aimed to address the question if maturation (i.e., men vs. boys) affects the physiological responses during repeated maximal sprints. Specifically, we compared men and preadolescent boys in terms of oxygen uptake and power output during repeated sprints, while adjusting for body size and mean power output (MPO). We also compared the effect of resistive load on these responses between men and boys. Based on all previous studies, it was hypothesized that the aerobic response would be greater in boys than men even when appropriately adjusting for body size differences.

## Materials and Methods

### Experimental Design

The present study compared oxygen uptake and MPO during repeated cycling sprints in men and preadolescent boys using heavier (optimum-Fopt) and lighter resistive loads (50% of Fopt). We have previously reported a significant effect of resistive loads on fatigue in children (Bogdanis et al., 2007). In this study, we compared if effects of resistive load are different between men and boys. The dependent variables examined in the present study were oxygen uptake, mean power output during a repeated sprint test, heart rate (HR), and lactate concentration, while the independent variables were maturation status (men vs. boys) and the resistive load (heavy vs. light). During pre-testing, a significant group difference was observed in lean leg volume, body height, and BM (Table 1). Accordingly, we partitioned out body mass using both the traditional ratio-scaling and scaling with the use of body mass as a covariate (ANCOVA) to enable child-adult comparisons. In particular, all dependent variables were compared between children and adults using: (a) VO_2peak_: (i) traditional ratio scaling for body mass and (ii) logarithmic values analyzed with ANCOVA using “body mass” and “peak power output” as covariates, (b) repeated sprints VO_2_: (i) absolute values, (ii) logarithmic values analyzed with ANCOVA using “body mass” as a covariate, and (iii) logarithmic values analyzed with ANCOVA using “mean power output” as a covariate, and (c) repeated sprints mean power output: (i) absolute values, (ii) logarithmic values analyzed with ANCOVA using “lean leg volume” as a covariate, and (iii) traditional ratio scaling for “lean leg volume.”

**Table 1:** Anthropometric characteristics.

	Men (*n* = 9)	Boys (*n* = 11)
Age (yrs)	21.7 ± 0.6	11.8 ± 0.6^*^
Body weight (kg)	74.2 ± 5.4	43.8 ± 6.0^*^
Body height (cm)	175.4 ± 6.0	152.3 ± 6.9^*^
Body fat (%)	12.6 ± 3.0	17.7 ± 3.3^*^
Lean leg volume (L)	7.6 ± 1.2	4.1 ± 0.7^*^
Leg volume (L)	9.3 ± 0.9	5.8 ± 1.0^*^

Values are presented as mean ± SD.

* Significantly different between men and boys; *p* < 0.05.

### Participants

Nine adult males (age: 21.7 ± 0.6 years) and 11 preadolescent boys (age: 11.8 ± 0.6 years) participated in this study. All participants were well-familiarized with the tests and equipment. Preadolescent boys were competitive swimmers, and adult participants were recreational athletes, both with at least 3 years of training experience. Prior to any testing, the participants and their parents were informed about the purpose of the study, and the procedures, requirements, and possible risks involved, before providing written informed consent. All procedures were according to the Helsinki Declaration and conformed to the standards of the local ethics committee. An experienced researcher evaluated children’s sexual maturity and pubertal development according to Tanner’s scale (Tanner, 1962) for pubic hair (*P*) and genitals (*G*) development (mode value; *P* = 2 and *G* = 2).

### Procedures

**Figure 1:**
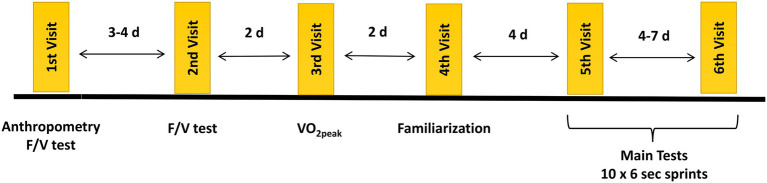
Schematic representation of study’s procedures. F/V test, force–velocity test.

During the first visit, participants performed a force-velocity test (Bogdanis et al., 2007) and completed anthropometry. The force-velocity test consisted of maximal cycle sprints at loads corresponding to 5, 7, and 9% of body mass (Bogdanis et al., 2007). During the second visit (3–4 days later), participants repeated the force-velocity test to get further familiarized with maximal sprinting. The test result from the second visit’s force-velocity was used to calculate the resistive load in the repeated cycle sprint test. Force-velocity data were analyzed as previously described (Arsac et al., 1996; Bogdanis et al., 2007), and the resistive load corresponding to the individual optimum force (Fopt) was determined. On the third visit, participants completed an incremental cycling test until exhaustion to determine peak oxygen uptake (VO_2peak_), while on the fourth visit, a familiarization trial was conducted. Participants completed seven consecutive 6-s sprints with 24-s of passive recovery against a resistive load corresponding to 75% of Fopt, to be thoroughly familiarized with the repeated sprints protocol. The main tests consisted of 10 maximal 6-s sprints separated by 24-s of passive recovery against two different resistive loads, performed in random and counterbalanced order 4–7 days apart. Participants sprinted against a heavy-load (Fopt) on one test occasion and a light-load (50% of Fopt) on another occasion. A summary of study’s procedures is presented in Figure 1.

### Anthropometry

Body fat percentage was estimated by measuring four skinfolds (biceps, triceps, subscapular, and suprailiac) and using age-specific equations (Durnin and Rahaman, 1967). Also, lean leg volume (LLV) was calculated to use as an indirect measure of lower body muscle mass used in cycling. LLV was determined by measuring leg lengths and circumferences and modeling the lower limb as six truncated cones (Jones and Pearson, 1969).

### Incremental Cycling Test

Peak oxygen uptake (VO_peak_) was measured using a standard incremental cycling test to exhaustion. The test was conducted on a modified friction-loaded cycle ergometer (Monark, model 864, Monark Exercise AB, Varberg, Sweden). VO_2_ data were collected using a breath-by-breath cardiopulmonary exercise test system (MedGraphics, CPX/D, United States). Before each test, analyzers were calibrated using calibration gases that covered the range of anticipated gas concentrations (one with a low O_2_ and high CO_2_ concentration and one with a high O_2_ concentration and zero CO_2_). The test duration was 10–12 min, and strong verbal encouragement was provided during the last minutes of the test to assist participants in reaching maximum values. The trial commenced at a power output of 35 W, which increased every minute for men (28 or 35 W) and boys (14 or 21 W) until exhaustion. Exhaustion was verified as previously described (Rowland et al., 2008), and specifically when at least three of the five following criteria were met: (1) plateau in oxygen uptake, (2) respiratory exchange ratio ≥ 1.1 for men and ≥1.0 for boys, (3) rate of perceived exertion (RPE) ≥ 17, (4) unable to maintain power output, and (5) HR which was within ±10 b/min (or 5%) of the age-predicted maximum (220-age in years). Once criteria were verified, peak VO_2_ was identified from the highest 30-s moving average.

### Repeated Sprints Tests

The repeated cycle sprint protocol consisted of 10 maximal 6-s sprints separated by 24-s of passive recovery against two different resistive loads (Fopt and 50% Fopt). Participants performed the protocol with heavy (Fopt) and light loads (50% Fopt) in random and balanced order 4–7 days apart. The load was individualized based on the previous force-velocity test. During the test, strong verbal encouragement was provided throughout the trial. The test was conducted on a modified friction-loaded cycle ergometer (Monark, model 864, Monark Exercise AB, Varberg, Sweden). The cycle ergometer was equipped with a restraining harness fitted around the participants’ waist to limit the movement to the lower limbs. Participants were instructed to achieve a maximal pedal rate as fast as possible from a standing start. A standardized general warm-up preceded the tests, consisting of 5 min cycling at 105 W for men and 70 W for boys. The specific warm-up consisted of two 30-s bouts at a high pedal rate at work-load corresponding to 50 W (100 rpm) and 60 W (120 rpm).

Mean power output, HR, and oxygen uptake were measured during each repeated sprint. Power output and pedal rate were measured and calculated as described earlier (Bogdanis et al., 2007) with the use of a photocell and a data acquisition system (MP100, Biopac Systems Inc., Goleta, United States). O_2_ and CO_2_ data for each sprint (6-s) were collected breath-by-breath (MedGraphics, CPX/D, United States) and interpolated at 1 Hz with a cubic spline interpolation program as previously described (Robergs and Burnett, 2003). Heart rate was measured after warm-up (pre HR), during the repeated sprint protocols, and post-exercise (3, 6, 9, and 12 min) with the use of a heart rate monitor (Polar Electro s610i, Kempele, Finland). Post-exercise HR recovery was determined by calculating the net change of HR at each time point (current HR—pre HR), expressed as a percentage of the difference between peak HR and pre HR. Peak HR was the highest HR value attained during the last sprint. Moreover, capillary arterialized blood samples were taken from a fingertip 3 min after warm-up and 3 min after completing the test. Lactate concentrations were measured using a micro-photometer (Dr Lange LP 20, Düsseldorf, Germany).

### Statistical Analysis

All statistical analyses were performed using SPSS statistical package (SPSS, v. 22, Chicago, IL). Normality was tested using the Kolmogorov–Smirnov test. Baseline measurements were compared between groups using a standard Students unpaired T-test. Previous studies have reported body mass as a cofounder when comparing peak VO_2_ between adults and children (Welsman et al., 1996). Therefore, we analyzed peak VO_2_ between boys and men with a univariate ANCOVA to adjust for body mass. Firstly, the logarithm of body mass, peak power output and peak VO_2_ were calculated. Secondly, the analysis was completed with “peak VO_2_” as the dependent variable, “age group” as the fixed factor, and “body mass” and “peak power output” as the covariates.

We used a repeated-measures design with one between-factor (group; men vs. boys) and two within factors (sprints and load) with and without covariates to compare boys and men VO_2_ and MPO produced during the repeated sprints. VO_2_ during repeated sprints was compared between children and adults using (i) absolute VO_2_ values (*post hoc* comparisons were conducted with the use of least significance difference method), (ii) VO_2_ logarithmic values analyzed with ANCOVA using “body mass” as a covariate, and (iii) VO_2_ logarithmic values analyzed with ANCOVA using the corresponding MPO for each sprint as a covariate. MPO during repeated sprints was compared between children and adults using (i) absolute MPO values, (ii) MPO logarithmic values analyzed with ANCOVA using “lean leg volume” as a covariate, and (iii) traditional ratio scaling for “lean leg volume” (VO_2_/L_LLV_).

Heart recovery data were analyzed using a three-way ANOVA with one between factor (group; men vs. boys) and two within factors (time and load). Post-exercise lactate response was analyzed with a two-way ANOVA, with one between factor (group; men vs. boys) and one within factors (load). We further examined significant effects with the least significant difference (LSD). In addition, we report effect size according to partial eta squared, which was considered small (~0.01), moderate (~0.06), or large (≥0.14). Pearson’s product–moment correlation was also used to determine the relation between MPO and body mass and LLV. The level of significance was set at *α* = 0.05. Results are presented as mean ± SD.

## Results

### Anthropometry

Anthropometrical data differed (*p* < 0.05) between men and boys (Table 1). Men were taller, had a greater body mass, a greater LLV, and a lower body fat percentage compared to boys (*p* < 0.05).

### Incremental Cycling Test

In absolute values, men had a greater peak VO_2_ than boys (Table 2) during the incremental cycling test (L min^−1^, *p* < 0.001, *η*^2^ = 0.81). However, no difference between age groups was noted when peak VO_2_ was presented as either ml kg^−1^ min^−1^ (*p* = 0.631, *η*^2^ = 0.012) or when body mass and power output were used as covariates in the analysis (body mass: *p* = 0.281, *η*^2^ = 0.068; vs. power output *p* = 0.100, *η*^2^ = 0.151). Thus, differences in peak VO_2_ between men and boys were mainly attributed to body size and power output differences.

**Table 2:** Peak oxygen uptake (VO_2_), peak power output (PPO), and peak VO_2_ adjusted for body mass (BM) and PPO in boys and men.

	Men (*n* = 9)	Boys (*n* = 11)	*p*
Peak VO_2_ (L min^−1^)	3.6 ± 0.4	2.2 ± 0.4	<0.001
Peak VO_2_ (ml kg^−1^ min^−1^)	48.9 ± 3.8	50.0 ± 6.4	0.631
Peak power output (W)	300.3 ± 25.3	183.5 ± 34.1	<0.001
Adjusted peak VO_2_ (ml min^−1^)_BM_	2933.6 ± 643.9	2578.6 ± 526.4	0.281
Adjusted peak VO_2_ (ml min^−1^)_PPO_	2887.1 ± 321.8	2614.9 ± 273.9	0.100

Values are presented as mean ± SD.

### Repeated Cycling Sprints

**Figure 2:**
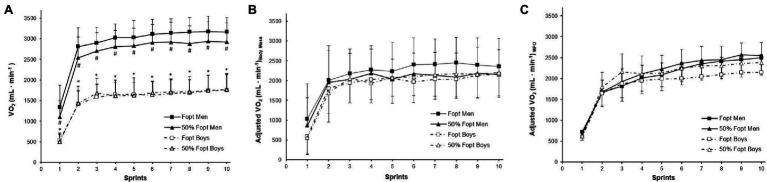
Oxygen uptake response to 10 repeated cycling sprints at light and heavy resistive loads in boys and men. VO_2_ in **(A)** absolute values, ml min^−1^, **(B)** adjusted for body mass, and **(C)** adjusted for mean power output (MPO). ^*^Significantly different between boys and men in the heavy-load condition; *p* < 0.05. ^#^Significantly different between boys and men in the light-load condition; *p* < 0.05.

In absolute values (ml min^−1^), VO_2_ in each repeated sprint was lower in boys than men in heavy-load (mean all 10 sprints: adults: 2887.4 ml min^−1^ vs. children: 1531.1 ml min^−1^, *p* = 0.05, *η*^2^ = 0.841) and light-load conditions (mean all 10 sprints: adults: 2656.1 ml min^−1^ vs. children: 1548.8 ml min^−1^, *p* = 0.003, *η*^2^ = 0.857; Figure 2A). There was a significant main effect for resistive loads (*p* = 0.047, *η*^2^ = 0.011), sprints (*p* < 0.001, *η*^2^ = 0.720), and group (*p* < 0.001, *η*^2^ = 0.696). There was also a significant load-by-group interaction effect (*p* < 0.0016, *η*^2^ = 0.016) but no sprints-by-group interaction (*p* = 0.185, *η*^2^ = 0.034). Introducing covariates in the model showed that both body mass (*B* = 0.942) and MPO (*B* = 0.738) were significant (*p* < 0.001) covariates (Tables 3, 4). Adjusting VO_2_ for body mass showed significant main effects for resistive load (*p* = 0.019, *η*^2^ = 0.015), sprints (*p* < 0.001, *η*^2^ = 0.784), and group (*p* = 0.007, *η*^2^ = 0.020) as well as significant interaction effects on load-by-group (*p* = 0.004, *η*^2^ = 0.023) and sprints-by-group (*p* = 0.041, *η*^2^ = 0.047). VO_2_ were comparable in men and boys during each repeated cycling sprint when adjusting for body mass (mean all 10 sprints: Heavy = 2173.9 ml min^−1^ for adults vs. 1876.2 ml min^−1^ for children and Light = 1981.6 ml min^−1^ for adults vs. 1910.6 ml min^−1^ for children; Figure 2B). Lastly, when adjusting VO_2_ for the corresponding MPO in each sprint, there were only main effects for load (*p* = 0.003, *η*^2^ = 0.024) and sprints (*p* < 0.001, *η*^2^ = 0.795). There was no main effect for group (*p* = 0.248, *η*^2^ = 0.004), no sprints-by-group interaction effect (*p* = 0.217, *η*^2^ = 0.032) and no load-by-group interaction effect (*p* = 0.078, *η*^2^ = 0.009). No child-adult differences were observed in VO_2_ when adjusting for the corresponding MPO in each sprint (mean all 10 sprints: Heavy = 2024.0 ml min^−1^ for adults vs. 1849.0 ml min^−1^ for children and Light = 2097.6 ml min^−1^ for adults vs. 2039.8 ml min^−1^ for children; Figure 2C).

**Table 3:** Parameter estimates and 95% CIs for the dependent variable, LnVO_2_ during the repeated sprint protocol when body mass was used as covariate.

Parameter	B	Std. Error	*p*	95% CI for B
Intercept	3.916	0.338	<0.001	3.252–4.580
LnBM	0.942	0.077	<0.001	0.790–1.094
Load	0.087	0.084	0.303	−0.079–0.252
Age	−0.017	0.090	0.853	−0.193–0.160

Ln, natural log; Std, standard; and BM, body mass. *R*^2^ = 0.882 (adjusted *R*^2^ = 0.869).

**Table 4:** Parameter estimates and 95% CIs for the dependent variable, LnVO_2_, during the repeated sprint protocol when mean power output (MPO) was used as covariate.

Parameter	B	Std. Error	*p*	95% CI for B
Intercept	3.266	0.409	<0.001	2.460–4.071
LnMPO	0.738	0.064	<0.001	0.613–0.863
Load	−0.023	0.086	0.790	−0.192–0.146
Age	−0.071	0.90	0.427	−0.248–0.105

Ln, natural log; Std, standard; and MPO, mean power output taken from each sprint. *R*^2^ = 0.879 (adjusted *R*^2^ = 0.866).

**Figure 3:**
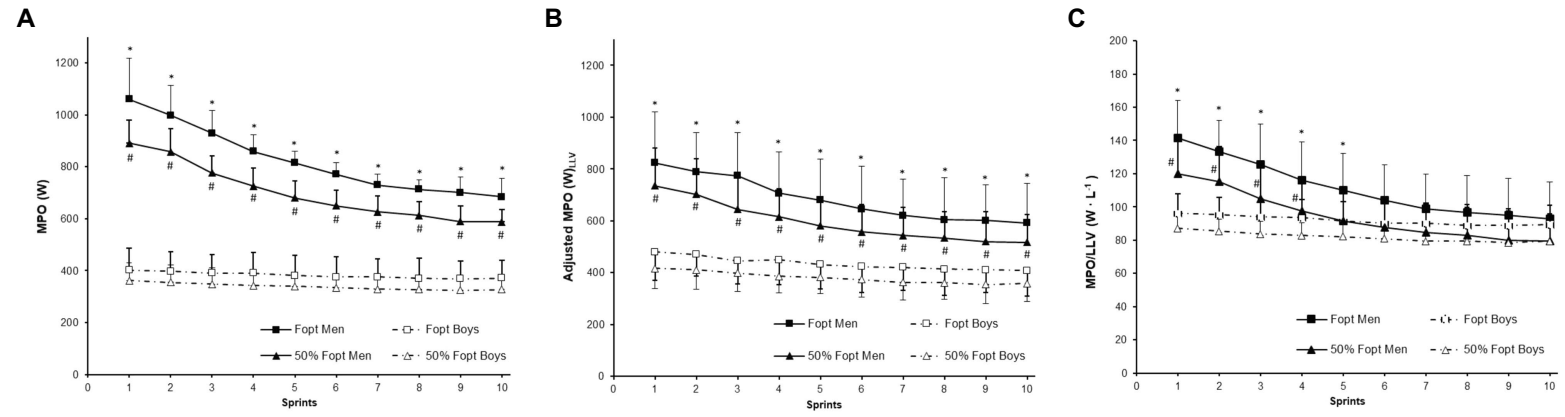
Mean power output in 10 repeated cycling sprints at light and heavy resistive loads in boys and men. MPO in **(A)** absolute values, W, **(B)** adjusted for LLV, and **(C)** conventional ratio-scaling (W L_LLV_^−1^). ^*^Significantly different between boys and men in the heavy-load condition; *p* < 0.05. ^#^Significantly different between boys and men in the light-load condition; *p* < 0.05.

Men produced higher mean power output (W) irrespective of load in all 10 sprints (Figure 3A; mean all 10 sprints: Heavy = 826.0 W for adults vs. 377.8 W for children, *p* < 0.001, *η*^2^ = 0.885, Light = 699.8 W for adults vs. 343.5 W for children, *p* < 0.001, *η*^2^ = 0.908). There were significant main effects for load (*p* < 0.001, *η*^2^ = 0.192), sprints (*p* < 0.001, *η*^2^ = 0.274), group (*p* < 0.001, *η*^2^ = 0.873), and a significant sprints-by-group interaction (*p* < 0.001, *η*^2^ = 0.135). LnLLV was used as a covariate in the model which showed a positive and significant scaling exponent (Table 5). When MPO was adjusted for the differences in LLV, there was a significant main effect for load (*p* < 0.001, *η*^2^ = 0.273), sprints (*p* < 0.001, *η*^2^ = 0.375), group (*p* < 0.001, *η*^2^ = 0.482), and a time-by-group interaction effect (*p* < 0.001, *η*^2^ = 0.199). Even after adjusting for LLV, men produced higher mean power output than boys in all sprints (mean all 10 sprints: Heavy = 683.9 W for adults vs. 435.5 W for children and Light = 594.9 W for adults vs. 380.8 W for children; Figure 3B). In contrast, conventional ratio-scaling (W·L_LLV_^−1^) showed higher relative MPO in men than boys (sprint × group interaction, *p* = 0.004, *η*^2^ = 0.855) only at the first five sprints (*p* < 0.05) at the heavy-load condition and the first four sprints (*p* < 0.05) at the light-load condition (Figure 3C).

**Table 5:** Parameter estimates and 95% CIs for the dependent variable, LnMPO, during the repeated sprint protocol when LLV was used as covariate.

Parameter	B	Std. Error	*p*	95% CI for B
Intercept	5.348	0.081	<0.001	5.188–5.508
LnLLV	0.509	0.035	<0.001	0.440–0.579
Load	0.149	0.056	0.009	0.259–0.019
Age	−0.287	0.058	<0.001	−0.174–0.065

Ln, natural log; Std, standard; MPO, mean power output taken from each sprint; and LLV, lean leg volume. *R*^2^ = 0.926 (adjusted *R*^2^ = 0.918).

There was strong correlation between MPO produced during the sprints and body mass (*r* = 0.883, *p* < 0.001) and LLV (*r* = 0.879, *p* < 0.001).

### Heart Rate and Blood Lactate

**Figure 4:**
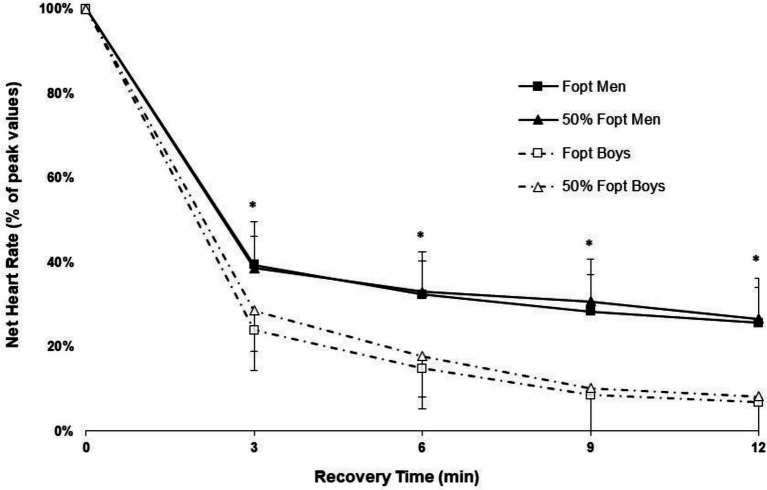
Heart rate (HR) recovery after 10 repeated cycling sprints at light (50% Fopt) and heavy resistive loads (Fopt) in boys and men. Values are net change of HR (current HR—pre HR), expressed as a percentage of the difference between peak HR and pre HR. ^*^Significantly different between boys and men in both conditions; *p* < 0.01.

Heart rate recovery data showed a significant group × time interaction (*p* = 0.026). Boys’ post-exercise HR recovery was significantly faster than men’s through all post-exercise measurements in both loading conditions (Figure 4).

Post-exercise blood lactate response was higher in men than boys after both resistive loads (*p* < 0.05). Men had a higher blood lactate response than boys after the repeated sprints at heavy-resistive load (men = 17.2 ± 5.0 mmol L^−1^ vs. boys = 9.9 ± 2.8 mmol L^−1^, *p* = 0.001, *η*^2^ = 0.49). Similarly, after repeated sprints at light resistive load, men’s blood lactate response was higher than boys’ (men = 18.2 ± 5.7 mmol L^−1^ vs. boys = 10.8 ± 2.6 mmol L^−1^, *p* = 0.004, *η*^2^ = 0.45).

## Discussion

The present study is the first to compare men’s and preadolescent boys’ oxygen uptake and power output production during repeated maximal sprints. The primary purpose was to test if children’s aerobic response is higher than adults during repeated sprints. The study’s main finding was that men’s MPO was higher than boys over the 10 repeated sprints, even when power output was adjusted for lean leg volume. However, in contrast to our hypothesis, we observed comparable oxygen uptake responses in men and boys over the 10 repeated sprints when appropriately adjusting for body mass or MPO. Furthermore, preadolescent boys showed a faster post-exercise heart rate recovery and had lower post-exercise blood lactate accumulation than men.

### Aerobic Response to Repeated Sprints

The present study is the first to compare oxygen uptake during repeated sprints in men and preadolescent boys. Contrary to our hypothesis, the VO_2_ response during repeated sprints was comparable between men and boys when appropriately adjusting for body mass or when adjusting for MPO. Previous research has suggested that children’s ability to resist fatigue is attributed to their greater muscle oxidative capacity, as indicated by a faster phosphocreatine resynthesis (Ratel et al., 2002, 2008). However, studies comparing aerobic energy system in adults and children are limited and conflicting. Some have compared muscle enzyme and fiber types in children and adults and suggested that children rely more on aerobic muscle metabolism (Eriksson et al., 1973). However, others have found no difference in type-I muscle fibers between adults and children (Bell et al., 1980). In addition, studies using the 31-phosphorus magnetic resonance spectroscopy method reported larger ATP regeneration capacity through aerobic mechanisms in children compared to adults (Ratel et al., 2008). Given the limited evidence and methodological difficulties, it is still unclear if children use more aerobic energy during repeated all-out short bouts.

A significant concern when comparing adults and children is the partitioning out or scaling for differences in body size (Van Praagh and Doré, 2002). Body size has previously been reported to explain the difference in submaximal VO_2_ between adults and children (Rogers et al., 1995), reflecting adults’ larger stroke volume, muscle mass, and blood volume. Our data showed a significantly lower oxygen uptake in children for all sprints in both resistive loads when absolute values were compared (Figure 2A). Other studies that measured VO_2_ during single bouts of high-intensity exercise reported that children have significantly greater ratio-scaled oxygen uptake than adults (Armon et al., 1991). In contrast, our data showed a comparable oxygen uptake in the repeated sprints between men and boys when appropriately adjusting for body mass (Figure 2B) or when adjusting for MPO (Figure 2C). We argue that the difference compared to previous studies (Armon et al., 1991) is due to the scaling method used since traditional ratio-scaling has been criticized for not adequately accounting for differences in body size (Welsman et al., 1996). The present study’s strength was the use of ANCOVA that allowed a fair child-adult comparison by adjusting for body mass differences (Nevill et al., 1992).

### Influence of Resistive Load on VO_2_ During Repeated Sprints

There was a significant main effect for load and age-by-load interaction for absolute VO_2_ during the repeated sprints (Figure 2A). Oxygen uptake in absolute values was higher in the heavy-load condition than the light-load condition, mainly driven by adults’ higher VO_2_ values on the heavy-load condition. However, when adjusting for the difference in MPO, child-adult differences disappeared (Figure 2C), and only main effect for load remained. The lack of age-by-group interaction in the adjusted means could be caused by adults’ larger muscle mass, which produces a greater power output. This is partly supported by our sample’s strong correlation between MPO and LLV (*r* = 0.879), suggesting that greater estimated leg muscle mass produces higher MPO.

Previous research (Dorel et al., 2003) has shown that a higher MPO produces higher total work during the repeated sprints, which results in higher VO_2_. Further, the efficiency/velocity relationships for type I and type II muscle fibers during cycling sprints have shown already from the 90’s that efficiency is higher when cycling against an optimum resistive load (Fopt) which corresponds to optimum pedal speed (Cherry et al., 1997). Our data showed a statistically significant main effect for resistive load (*p* = 0.003) even when VO_2_ was adjusted for the MPO produced in each sprint (Figure 2C). Interestingly, the effect of the resistive load was different in absolute VO_2_ and VO_2_ adjusted for MPO. Heavy-load produced higher VO_2_ than light-load during absolute VO_2_, and in contrast, light-load produced higher VO_2_ values when adjusted for MPO. The increased VO_2_ observed in the light-load condition when adjusting for MPO highlights the significance of efficiency. More specifically, the pedal speed during sprinting against light load is higher than the optimum pedal speed corresponding to Fopt. Higher pedal speed increases muscle contractions and movements, which increases the energetic cost for sprinting (Belli and Hintzy, 2002). This is reflected in higher adjusted VO_2_ values during sprinting against light loads.

### Power Output in Repeated Sprints

Power output is highly influenced by body size (Van Praagh and Doré, 2002), and in order to partition out the difference in body size, we used lean leg volume rather than body mass. Lean leg volume was used as a scaling variable since scaling for active muscle mass is more appropriate for non-weight-bearing exercises like cycling (Doré et al., 2005). The commonly used scaling method is to divide power output with lean leg volume (ratio-scaling). Ratio-scaling has been reported to be an ineffective method for scaling for body size and produce misleading child-adult comparison (Santos et al., 2003). Therefore, the use of regression standards and analyses of covariance (ANCOVA) has been recommended (Doré et al., 2005).

In line with previous research (Santos et al., 2002), our study showed a difference between conventional ratio-scaling and adjusted power output. Consistent with other studies (Ratel et al., 2004), our data showed significantly higher ratio-scaled mean power output in men than boys only for the first repeated sprints but not the latter. Since ratio-scaling does not effectively partition out LLV, we also analyzed the data with ANCOVA to adjust for lean leg volume differences. When mean power output was adjusted by lean leg volume, adults produced significantly higher power output in all 10 sprints, irrespective of loading condition. These findings are consistent with previous research showing that conventional ratio-scaling possibly masks an age-related effect (Santos et al., 2003). It seems that other physiological mechanisms such as children’s lower glycolytic and enzymatic activity (Ratel et al., 2006) may also contribute to the observed differences. In addition, some have suggested that children likely possess higher type-I muscle fiber composition (Lexell et al., 1992), although research is not consistent (Bell et al., 1980). A higher type-I muscle fiber is associated with aerobic activities and may partially explain why children’s power output is significantly lower than adults. Others have proposed that child-adult muscle function differences are due to children’s inability to recruit or effectively use type-II motor units (Dotan et al., 2012).

### Heart Rate and Blood Lactate Responses

Faster heart rate recovery has been reported in preadolescent children compared to adults following short high-intensity exercise (Hebestreit et al., 1993; Birat et al., 2018). Heart rate recovery in the present study is in line with previous research showing a faster recovery in preadolescent boys following repeated sprints, irrespective of the load used. Possible reasons for a faster heart rate recovery in children are currently not fully understood. One explanation could be that children’s bodies are smaller than adults, resulting in shorter cardiovascular circulation distances and more rapid circulation (Falk and Dotan, 2006).

Further, post-exercise lactate was significantly higher in men compared to boys. A higher peak lactate response following high-intensity exercise in adults is typically reported in the literature (Ratel et al., 2002, 2004). Differences in lactate responses can partially be explained by quantitative differences between children and adults. More specifically, lactate production is related to intensity, and since adults produce higher total work during high-intensity repeated exercise, it leads to higher concentrations of blood lactate compared to children. Another possible explanation might be children’s lower ability to activate type-II motor units and thus produce lactate (Falk and Dotan, 2006). If maturation affects, the motor unit recruitment pattern is not well understood (Dotan et al., 2012). A metabolic profile with a higher percentage of type-I muscle fibers would, in theory, explain why children produce lower power output and less lactate.

## Limitations

The main limitation of the present study is the small sample size which limits its external validity. The fact that there were no differences in VO_2_ might result from the small sample size that has led to a type II error. However, the small effect sizes suggest that it is doubtable that a larger number of participants could lead to statistically significant differences between children and adults. In addition, the study’s external validity is further limited because all participants were trained individuals. Prepubertal boys were competitive swimmers, and they were selected because they were familiar with maximal performance, while men were recreational athletes and had previously been competitive athletes. Participants’ training background decreases the applicability of the results in the general population. Despite this, our study provides novel information regarding differences in oxygen uptake during repeated sprints between children and adults.

## Conclusion

In conclusion, the present study provided no evidence of a higher VO_2_ response in boys compared to men in repeated sprints when appropriately scaled for body mass or MPO. Despite the faster HR recovery and the lower post-exercise lactate concentrations, adjusted VO_2_ response of the present study does not support the hypothesis of a higher aerobic response in children during repeated sprints. Additionally, men’s power output was significantly greater than boys in repeated sprints, even when appropriately scaled for lean leg volume. The present study further highlights the importance of using appropriate scaling methods when comparing adults and children.

## Data Availability Statement

The raw data supporting the conclusions of this article will be made available by the authors, without undue reservation.

## Ethics Statement

The studies involving human participants were reviewed and approved by National and Kapodistrian University of Athens. Written informed consent to participate in this study was provided by the participants’ legal guardian/next of kin.

## Author Contributions

AT, GB, AP, and MM designed the present study. AT and AP collected the data. AT, GB, DJ, and AN analyzed the data. AT, GB, DJ, and MM undertook the data interpretation. AT and DJ contributed to the manuscript preparation. All authors contributed to the article and approved the submitted version.

## Conflict of Interest

The reviewer AZ declared a past collaboration with one of the authors GB to the handling editor.

The remaining authors declare that the research was conducted in the absence of any commercial or financial relationships that could be construed as a potential conflict of interest.

## Publisher’s Note

All claims expressed in this article are solely those of the authors and do not necessarily represent those of their affiliated organizations, or those of the publisher, the editors and the reviewers. Any product that may be evaluated in this article, or claim that may be made by its manufacturer, is not guaranteed or endorsed by the publisher.
